# Simultaneous Effect of Temperature and Irradiance on Growth and Okadaic Acid Production from the Marine Dinoflagellate *Prorocentrum belizeanum*

**DOI:** 10.3390/toxins6010229

**Published:** 2014-01-06

**Authors:** Lorenzo López-Rosales, Juan Jose Gallardo-Rodríguez, Asterio Sánchez-Mirón, María del Carmen Cerón-García, El Hassan Belarbi, Francisco García-Camacho, Emilio Molina-Grima

**Affiliations:** Area of Chemical Engineering, University of Almería, Almería 04120, Spain; E-Mails: llr288@ual.es (L.L.-R.); jgr285@ual.es (J.J.G.-R.); asmiron@ual.es (A.S.-M.); mcceron@ual.es (M.C.C.-G.); ebelarbi@ual.es (E.H.B.); emolina@ual.es (E.M.-G.)

**Keywords:** dinoflagellate, microalga, irradiance, temperature, photobioreactor, *Prorocentrum belizeanum*, modelling

## Abstract

Benthic marine dioflagellate microalgae belonging to the genus *Prorocentrum* are a major source of okadaic acid (OA), OA analogues and polyketides. However, dinoflagellates produce these valuable toxins and bioactives in tiny quantities, and they grow slowly compared to other commercially used microalgae. This hinders evaluation in possible large-scale applications. The careful selection of producer species is therefore crucial for success in a hypothetical scale-up of culture, as are appropriate environmental conditions for optimal growth. A clone of the marine toxic dinoflagellate *P. belizeanum* was studied *in vitro* to evaluate its capacities to grow and produce OA as an indicator of general polyketide toxin production under the simultaneous influence of temperature (*T*) and irradiance (*I*_0_). Three temperatures and four irradiance levels were tested (18, 25 and 28 °C; 20, 40, 80 and 120 µE·m^−2^·s^−1^), and the response variables measured were concentration of cells, maximum photochemical yield of photosystem II (PSII), pigments and OA. Experiments were conducted in T-flasks, since their parallelepipedal geometry proved ideal to ensure optically thin cultures, which are essential for reliable modeling of growth-irradiance curves. The net maximum specific growth rate (*µ_m_*) was 0.204 day^−1^ at 25 °C and 40 µE·m^−2^·s^−1^. Photo-inhibition was observed at *I*_0_ > 40 μEm^−2^s^−1^, leading to culture death at 120 µE·m^−2^·s^−1^ and 28 °C. Cells at *I*_0_ ≥ 80 µE·m^−2^·s^−1^ were photoinhibited irrespective of the temperature assayed. A mechanistic model for *µ*_m_-*I*_0_ curves and another empirical model for relating *µ*_m_-*T* satisfactorily interpreted the growth kinetics obtained. ANOVA for responses of PSII maximum photochemical yield and pigment profile has demonstrated that *P. belizeanum* is extremely light sensitive. The pool of photoprotective pigments (diadinoxanthin and dinoxanthin) and peridinin was not able to regulate the excessive light-absorption at high *I*_0_-*T*. OA synthesis in cells was decoupled from optimal growth conditions, as OA overproduction was observed at high temperatures and when both temperature and irradiance were low. T-flask culture observations were consistent with preliminary assays outdoors.

## 1. Introduction

Microalgal dinoﬂagellates are a major source of structurally diverse bioactive natural products and toxins with potential uses in biomedical, toxicological and pharmacological research [1]. Most of these molecules have not progressed beyond the discovery stage because of limited availability from the natural source and difﬁculty of synthesis due to structural complexity. Many source species have proved difﬁcult to culture in the laboratory or have failed to produce the desired compound under artiﬁcial culture conditions. In fact, of the marine bioactives and toxins under clinical evaluation [2], none has been sourced from dinoﬂagellates. Toxins from dinoﬂagellates are also attracting increasing attention because of their impact on the safety of seafood. 

Only sparing quantities of dinoﬂagellate toxins are generally available, and this hinders bioactivity characterization and evaluation in potential applications. Approaches to production of increased quantities of dinoﬂagellate bioactives are needed [3], since the titer of the target toxin is often low. For example, the production of 150 g of a certain dinoflagellate toxin is estimated to require a culture broth volume of between 10^3^ (*Alexandrium minute*) and 10^5^ m^3^ (*Gambierdiscus toxicus*) [4]. This is far larger than the volume of the fermentation broth needed to produce the same quantity of a typical antibiotic, for example. The handling of such a large volume of broth of a highly toxic material could be a major problem [3]. Raising the concentration of the bioactive in the broth is a major issue to reduce both the volume to be handled and production costs. The concentration of a target compound can be enhanced by modifying and combining different levels of culture of abiotic factors. 

Under nutrient excess, light and temperature are widely accepted as the driving factors acting on the overall biochemical composition of biomass and growth in microalgal cultures. Laboratory studies have also confirmed that temperature and light can significantly influence the growth rate and toxin production of dinoflagellates [5,6]. It is therefore essential to establish experimentally the individual and/or interacting effects of irradiance and temperature on cultures of dinoflagellates of interest in order to improve growth and toxin yields. 

The marine dinoflagellate *Prorocentrum belizeanum* was selected because it is a recent source of okadaic acid analogues, polyketides and macrolides of interest (e.g., corozalic acid, belizeanolide, belizeanic acid) [7,8,9,10,11,12]. Marine dinoflagellates of the genus *Prorocentrum* are famous for the production of okadaic acid (OA) and its analogues, which are inhibitors of protein phosphatases types 1 (PP1) and 2A (PP2A), and causative toxins of diarrhetic shellfish poisoning (DSP) [13].

Since the physiology of *P. belizeanum* is yet to be investigated as a required stage of a potential bioprocess, the aim of this work was to evaluate the simultaneous influence of irradiance and temperature on growth kinetics, maximum photochemical yield of the photosystem II (PSII), pigment profile and OA production, by using several combinations of the two variables. This work also sought to establish relationships between response variables and the physiological state of the cells, in order to monitor more easily the current status of the culture.

## 2. Results and Discussion

### 2.1. Growth Kinetics and Modeling

The relative attenuation of irradiance in the T-Flask cultures over time was calculated as detailed in the Materials and Methods section. In general, the mutual shading increased with the age of the culture and the cell concentration (data not shown). The highest light attenuation values were observed in the stationary phase, reaching a maximum of around 10% in the experiment carried out at 25 °C and 40 µE·m^−2^·s^−1^. In the exponential phase, mutual shading in all the cultures was substantially below 10%. Consequently, our cultures can be considered optically thin, *i.e.*, it is unlikely that self-shading affected the experimental results reported. This issue is critical in the modeling of the *P*-*I* curves (growth or photosynthesis rate versus irradiance). These curves derived from laboratory studies are commonly used to predict biomass productivity in photobioreactors or natural habitats (e.g., microalgal blooms), where the cell density is high, and hence also the effect of light attenuation. If the estimated kinetic parameters are markedly affected by mutual shading or acclimation phenomena, the predictions could be wrong, and the denser the culture systems, the greater the error. Novel aspects related with the modeling of *P-I* curves in microalgae have comprehensively been addressed in a recent study [14]. 

**Figure 1:**
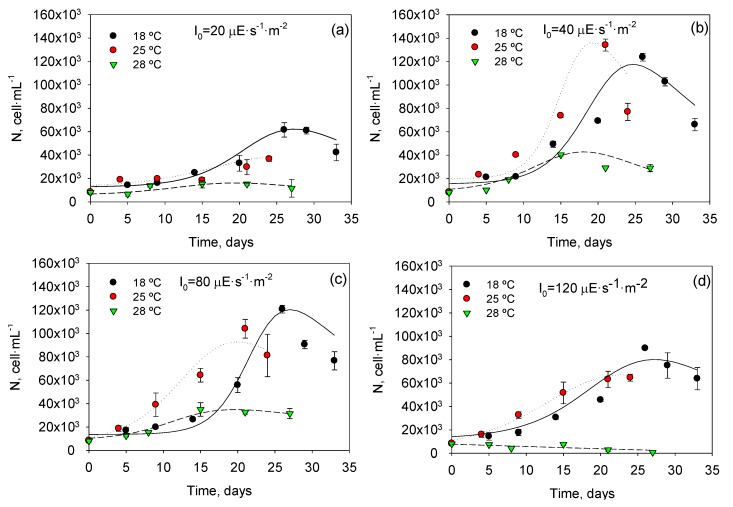
Temporal variation of the cell concentration of *Prorocentrum belizeanum* grown in T-flask batch cultures at the different irradiances and temperatures assayed: (**a**) 20 µE·m^−2^·s^−1^; (**b**) 40 µE·m^−2^·s^−1^; (**c**) 80 µE·m^−2^·s^−1^; (**d**) 120 µE·m^−2^·s^−1^. Solid lines are the fits obtained from Equation (2). Experimental data are given as the average of duplicate cultures ± SD.

**Figure 2:**
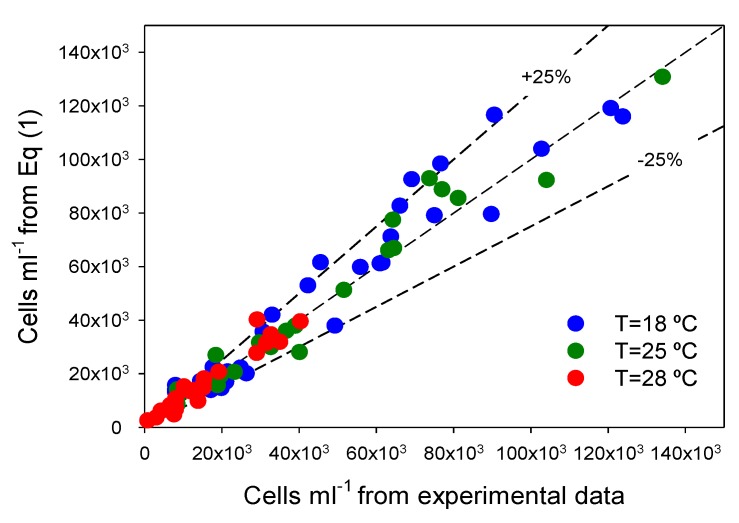
Illustration of the ability of the asymmetric logistic equation (Equation (2) of Experimental Section 3) to fit experimental data. Experimental data for all experiments are compared with Equation (2) predictions. Every temperature gathers all the results for the four irradiance levels assayed. Diagonal represents an exact agreement.

Figure 1 shows profiles of cell density with time for all experiments and *T* and *I_0_* combinations. A maximum cell concentration of 134 × 10^3^ cells∙mL^−1^ was attained at 25 °C and 40 μEs^−1^m^−2^. It can also be observed that the selected asymmetric logistic equations (ALEs; see Experimental Section 3) were able to fit the different experimental asymmetric growth curves. As shown in Figure 2, there is a good agreement (within ±25%) between measured data and values predicted from ALEs for all datasets. Clearly, Equation (2) fits experimental data obtained under different *T* and *I*_0_ quite well, supporting its use for estimating the typical physiological response in all phases of *P. belizeanum* batch cultures.

**Figure 3:**
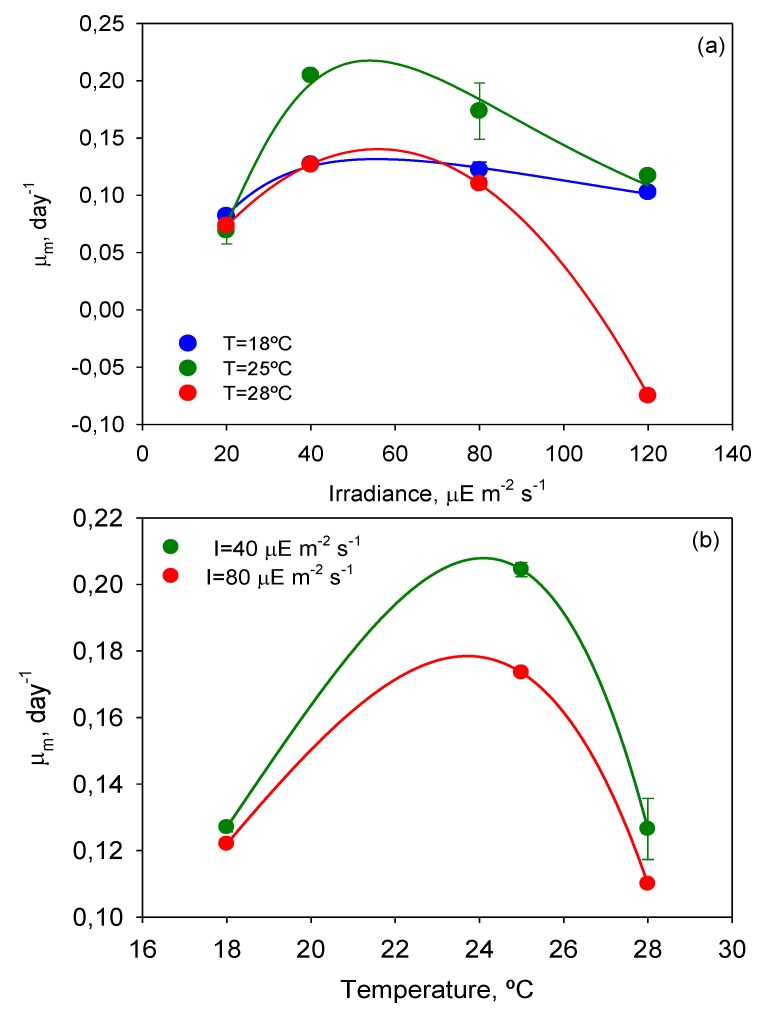
Maximum specific growth rate of *Prorocentrum belizeanum* as a function of irradiance level and temperature. Lines represent the estimated values from the fit of Equation (4) (**a**) and Equation (5) (**b**) to the experimental data. Experimental data are given as the average of duplicate cultures ± SD.

Maximum specific growth rates over time were calculated with Equation (3) by analytical differentiation of the ALE (Equation (2) of Experimental Section 3). The *µ*_m_ values of every T-Flask culture were used to evaluate the effect of *T* and *I*_0_ on cell growth. Inspection of Figure 3, where the dependence of *µ_m_* on *I*_0_ and *T* is illustrated, reveals that the temperature of 25 °C also provided the highest *µ_m_* value (0.204 day^−1^). Although no temperature effects on growth of *P. belizeanum* have been reported in the literature, optimal growth temperatures for other species of the genus *Prorocentrum* ranging from 10 to 29 °C are well-documented [15,16,17,18]. The optimal irradiance, *I*_opt_, seemed to be around 50 µE·m^−2^·s^−1^ at 25 °C. While this value is very close to the irradiance levels used in many studies to grow the genus *Prorocentrum* for different purposes [19,20,21,22,23], it is far lower than those reported as optimum for other species of the same genus. For example, *I*_opt_ for *Prorocentrum lima* [24,25] was found to vary from 150 to 180 µE·m^−2^·s^−1^. 

The curves *µ_m_*-*I*_0_ in Figure 3a are similar to the typical *P-I* curves of microalgae where three main areas can be distinguished: (i) a light-limited region (≤40 μEm^−2^s^−1^) in which the light-absorption step controls the rate of the subsequent cell metabolic processes; (ii) zone of higher illumination (around 50 μEm^−2^s^−1^) in which sufficient energy is available for the cell metabolic processes to become saturated and the rate of photosynthesis is no longer a strong function of the irradiance (*i.e.*, metabolic assimilation of energy becomes the rate-controlling step); and (iii) a photo-inhibition region (>40 μEm^−2^s^−1^), in which irradiance becomes detrimental, decreasing the growth rate and even leading to culture death as in the experiment carried out at *I* = 120 µE·m^−2^·s^−1^ and *T* = 28 °C. *P. belizeanum* cultures at *I*_0_ ≥ 80 µE·m^−2^·s^−1^ were photoinhibited irrespective of the temperature assayed. The extension and shape of the aforementioned *µ_m_*-*I*_0_ curve zones depended on the culture temperature (see Figure 3a). Microalgae can acclimatize their photosynthetic system in response to shifts in temperature, modifying their irradiance requirements for optimal growth. As a result, microalgae can tolerate higher irradiances at temperatures closer to their optimum [26], *i.e.*, saturation intensity increases with temperature. As stated above, and as Figure 3 illustrates, *P. belizeanum* presented an optimal growth temperature of around 25 °C, which corresponded to the highest *µ_m_* values. 

Lines in Figure 3 show the fit of the present models (Equations (4) and (5) of Experimental Section 3) to the experimental data of *µ*_m_. The best-fit values of the kinetic parameters are displayed in Table 1 and Table 2. The values of the parameters of the *µ*_m_-*I* model (see Table 1) were consistent with other simulations reported elsewhere [27]. For instance, the values of *α* reasonably correspond to the boundary irradiance value (*I*_0_ = *α*) that separates the light-limitation region from the light-saturated photosynthesis zone (see Figure 3a). Thus, when *I*_0_ < *α*, the photosynthesis or growth rate depends strongly on irradiance. When *I*_0_ > *α*, cultures are light-saturated, and if *I*_0_ is sufficiently increased above *α*, cells may become photoinhibited. As expected, values of the cell maintenance rate (*m*) were clearly lower than the maximum specific growth rate (*µ*_max_). Values of *µ*_max_ were similar. There is a degree of debate regarding the ideal value of exponent *n*, reaction order of the photoinhibition rate, which is 0.5 or 1 according to different authors [27]. The *n* values obtained were close to 1, the main deviation corresponding to 28 °C.

**Table 1:** Parameter estimation from fitting the µm-I model (Equation (4) of Experimental Section 3) to data gathered by temperature (see Figure 3a) using non-linear regression.

Parameter	Temperature		
	18 °C	25 °C	28 °C
*α* (µE m^−2^ s^−1^)	78.21	54.72	82.97
*κ* (-)	0.8545	0.0804	0.0000
*δ* (µE^−n^ m^2n^ s^n^)	4.6 × 10^−3^	1.3 × 10^−3^	3.7 × 10^−5^
*n* (-)	1.175	1.457	2.5
*m* (day^−1^)	0.046	0.152	0.143
*µ_max_* (day^−1^)	0.741	0.712	0.801
* *R*^2^ (-)	0.999	0.999	0.939

Note: * Determination coefficient.

**Table 2:** Parameter estimation from fitting the *µ*_m_-*T* model (Equation (5) of Experimental Section 3) to data gathered by irradiance (see Figure 3b) using non-linear regression.

Parameter	Irradiance	
	40 µE·m^−2^·s^−1^	80 µE·m^−2^·s^−1^
*T_min_* (°C)	0.00	0.00
*T_max_* (°C)	29.89	30.15
β (-)	6 × 10^−7^	1.3 × 10^−6^
γ (-)	4.16	3.68
*µ_max_* (day^−1^)	0.108	0.189
* *R^2^* (-)	0.999	0.999

Note: * Determination coefficient.

The values of the parameters for the *µ*_m_-*T* model in Table 2 were consistent with those reported by Huang *et al*. (2011) for microorganisms [28]. Other models based on the Arrhenius equation [29] could also have been suitable for fitting the data with similar accuracy within the experimental range (data not shown). However, the *T*_min_ values estimated by the Huang’s model (Equation (5) of Experimental Section 3) are closer to the biological minimum of both psychrotrophs and mesophyles as discussed elsewhere [28]. Even so, all the commonly used *µ*_m_-*T* models are formulated in such a way that *µ*_m_ has to be zero out of the interval [*T*_min_, *T*_max_]. It is an important hurdle because negative net growth rates cannot be predicted. In fact, experimental *µ*_m_-*T* data at 28 °C could not be fitted to Equation (5), since the *µ*_m_ value at 120 µE·m^−2^·s^−1^ was negative (see Figure 3a). Therefore, negative specific growth rates due to cell mortality should be taken into consideration for *T* > *T*_max_. This is critical in the control of outdoor microalgal production processes, in which biomass destruction after a high temperature episode (*T* > *T*_max_) should be taken into account for an accurate prediction of the total biomass evolution [30]. 

Modeling of the simultaneous effect of *T* and *I*_0_ on *µ*_m_ was not presented here, although it was relatively straightforward from Equations (4) and (5) by coupling the effects of *I*_0_ and *T*. From a realistic point of view, uncoupled models can be seen as a functional artefact to replace coupled models that better represent the interdependent impact of *T* and *I*_0_ on photosynthesis, but are more difficult to obtain. This *T-I*_0_ interplay should be expressed by variation of the kinetic parameters of any *P*-*I* model with the temperature. Variation of the parameters in Table 1 with *T* are evidence of this, as are other cases reported in the literature, e.g., variation of saturation irradiance with *T* in cultures of harmful dinoflagellates of the genus *Ceratium* [31]. However, coupled models have a large number of parameters, and therefore a high number of experiments are needed in order to fit without risk of overfitting [32]. Future work should focus on this direction. 

### 2.2. Maximum Photochemical Yield (F_v_/F_m_)

**Figure 4:**
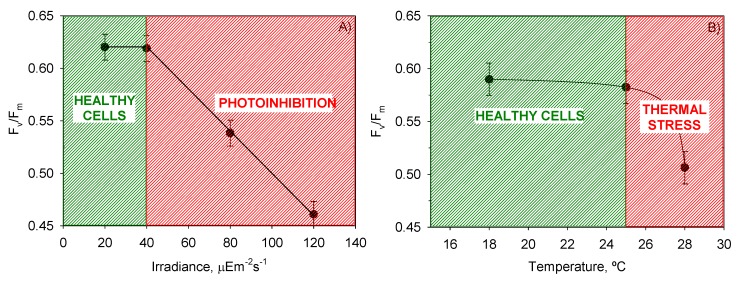
Evaluation from the ANOVA of the effect of (**A**) *I*_0_ and (**B**) *T* on the mean maximum photochemical yield of the PSII (F_v_/F_m_) of *P. belizeanum*. Every irradiance and temperature gathers all the results obtained for the three *T* and four *I*_0_ levels, respectively. Bars around points represent 95.0% confidence intervals based on Fisher’s least significant difference procedure. Overlapping bars indicate no significant difference.

*F*_v_/*F*_m_, which is the ratio of variable fluorescence to maximal fluorescence (see Experimental Section 3), provides an estimate of the PSII maximum efficiency within dark-adapted material. Measurements of *F*_v_/*F*_m_ with the time of culture were recorded for all the experiments. To analyze the effect of the factors involved (*T*, *I*_0_, and time) and their interactions on the variability of *F*_v_/*F*_m_, a multifactor ANOVA was carried out, and the results are shown in Table 3. All factors and interactions, except for *I*_0_-t, had a statistically significant effect on *F*_v_/*F*_m_ at the 95.0% confidence level (*p* < 0.05). However, the contributions of the time of culture (4.44%) and of the sum of all interactions (14.87%) to the global variation of *F*_v_/*F*_m_ were substantially lower than those of temperature (26.59%) and irradiance (54.09%). Figure 3 shows the mean *µ*_m_ values reached for each *T* and *I*_0_ and the intervals around each mean value. Fisher’s least significant difference (LSD) procedure was used for establishing discrimination among the means. Thus, for irradiances and temperatures above 40 µE·m^−2^·s^−1^ and 25 °C, respectively, there were statistically significant differences between factor levels. These values of *I*_0_ and *T* divided the plots in Figure 4 into two zones that allowed the separation of photoinhibited or thermally stressed cells from healthy cells in function of the recorded *F*_v_/*F*_m_. Photoinhibition of photosynthesis was clearly identified in Figure 4a by a light-induced depression in *F*_v_/*F*_m_. Photoinhibition at sub-optimal temperature (18 °C) was not observed in Figure 4b.

**Figure 5:**
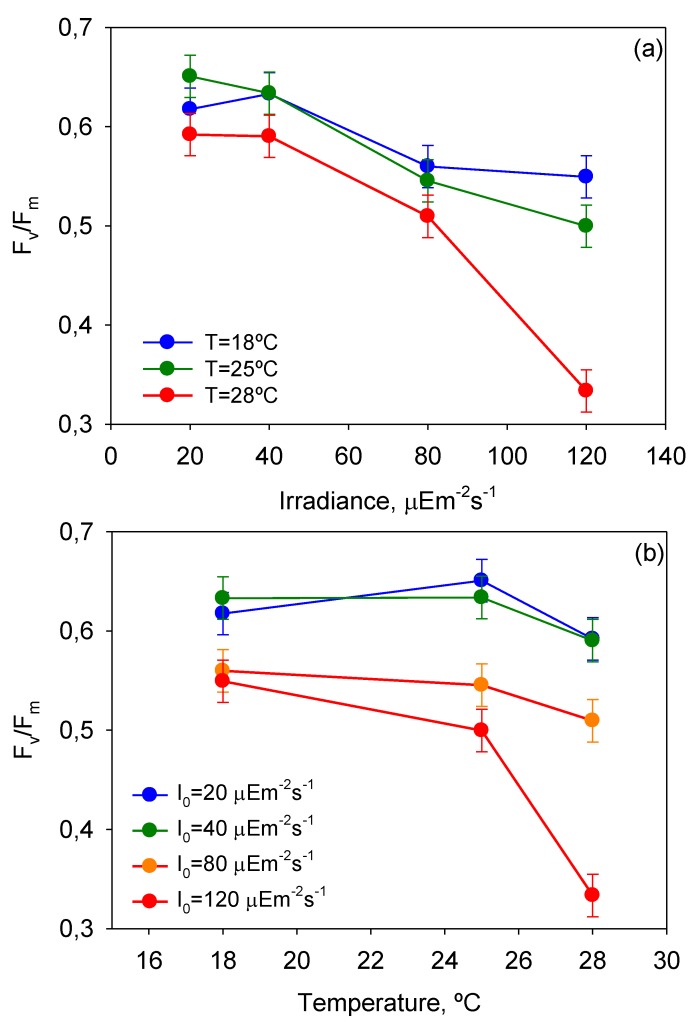
Plots of ANOVA interactions (**a**) *T*-*I*_0_ and (**b**) *I*_0_-*T* in the response of mean maximum photochemical yield of the PSII (*F*_v_/*F*_m_) of *P. belizeanum* (if the factors do not interact, the lines on the plot should be approximately parallel. If they are not, then the effect of one factor depends upon the level of the other). Every irradiance and temperature gathers all the results obtained for the three *T* and four *I*_0_ levels, respectively. Bars around points represent 95.0% confidence intervals based on Fisher’s least significant difference (LSD) procedure. Overlapping bars indicate no significant difference.

Figure 5 displays the impact of *T*-*I*_0_ interactions on *F*_v_/*F*_m_. Notice that the effect of photoinhibition and thermal stress is strengthened at high temperatures (>25 °C) and irradiances (>40 µE·m^−2^·s^−1^), respectively. These results are therefore in line with the aforementioned observations derived from the analysis of *µ*_m_-*I*_0_ and *µ*_m_-*T* curves.

**Table 3:** Multifactor ANOVA testing the effect of temperature (*T*), irradiance (*I*_0_) and time of culture (*t*) on different cellular responses: maximum quantum yield (*F*_v_/*F*_m_), cell content of pigments (Chlorophyll *a*, Chl *a*; Chlorophyll *c*_2_, Chl *c*; Peridinin, *Pe*; Diadinoxanthin, *Dd*; Dinoxanthin, *Dn*) and production of okadaic acid (OA). Overall variability is divided into several components: a component attributable to the main effect of each factor and another one attributable to the interaction between different factors. Percentage of variation of the response (Variation, %), explained by the contribution of each factor and interaction, as the percentage of the *F*-ratio of each factor relative to the sum of all F-ratios.

Response	Statistics	Main Effects			Interactions		
		*T*	*I*_0_	*t*	*T-I*_0_	*T-t*	*I*_0_-*t*
*F*_v_/*F*_m_	Variation (%)	26.59	54.09	4.44	5.15	6.62	3.10
	*p*-value	<0.05	<0.05	<0.05	<0.05	<0.05	>0.05
Ch*a* (pg cell^−1^)	Variation (%)	61.52	27.08	6.86	2.35	(-)	2.18
	*p*-value	<0.05	<0.05	<0.05	>0.05	(-)	>0.05
Ch*c* (pg cell^−1^)	Variation (%)	64.33	24.68	6.34	0.84	(-)	0.67
	*p*-value	<0.05	<0.05	>0.05	>0.05	(-)	>0.05
*Pe* (pg cell^−1^)	Variation (%)	68.09	15.01	6.77	5.65	(-)	4.46
	*p*-value	<0.05	<0.05	>0.05	>0.05	(-)	>0.05
*Dd* (pg cell^−1^)	Variation (%)	65.36	18.06	11.09	4.67	(-)	0.79
	*p*-value	<0.05	<0.05	<0.05	>0.05	(-)	>0.05
*Dn* (pg cell^−1^)	Variation (%)	17.64	52.24	4.27	16.85	(-)	8.97
	*p*-value	<0.05	<0.05	<0.05	<0.05	(-)	<0.05
*OA* (pg·cell^−1^)	Variation (%)	79.02	16.94	(-)	4.04	(-)	(-)
	*p*-value	<0.05	<0.05	(-)	<0.05	(-)	(-)
*OA* (ng·mL^−1^)	Variation (%)	98.92	1.084	(-)	0.48	(-)	(-)
	*p*-value	<0.05	<0.05	(-)	<0.05	(-)	(-)

**Figure 6:**
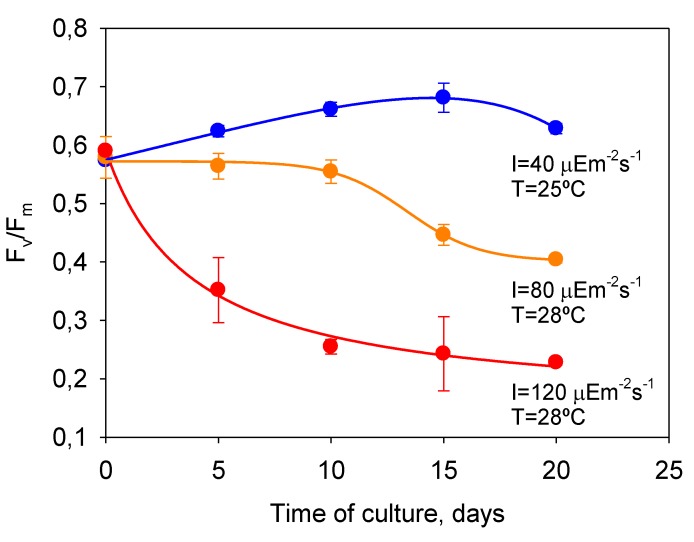
Temporal variation of dark-adapted *F*_V_/*F*_m_ in cultures of *P. belizeanum* exposed to experimental conditions with different stress levels. Data points are averages, and vertical bars are standard errors of the means.

Microalgal cultures with *F*_v_/*F*_m_ values ranging from 0.6 for cyanobacteria to 0.8 for green algae are generally considered healthy by algologists. Although to date there is no established consensus about dinoflagellates, *F*_v_/*F*_m_ values of around 0.6 also seem to be indicative of healthy cells (see Figure 4). Depending on the growth conditions, *F*_v_/*F*_m_ can vary significantly during cultivation. Hence a decline in *F*_v_/*F*_m_ from optimal values represents a reliable warning indicator for preventing or treating stressed microalgal culture as reported elsewhere [33]. Dinoflagellates can also experience sharp declines in *F*_v_/*F*_m_ under prolonged exposure to thermal and/or light stress [34,35,36]. *P. belizeanum* showed a similar behavior, as Figure 6 shows. In the severely photoinhibited and thermally stressed culture (28 °C and 120 µE·m^−2^·s^−1^), *F*_v_/*F*_m_ markedly decreased from 0.65 for unstressed conditions at the beginning of the culture, to below 0.3 after 10 days of culture, indicating a strong photoinactivation of the PSII. *F*_v_/*F*_m_ in the culture under optimal *T* and *I*_0_ (25 °C and 40 µE·m^−2^·s^−1^) showed consistent values from 0.6 to 0.7 by contrast. In the culture moderately photoinhibited (*T* = 28 °C and 80 µE·m^−2^·s^−1^), *F*_v_/*F*_m_ only began to significantly decline from day 10. 

The analysis of the results presented in this section showed that the *F*_v_/*F*_m_ measurements are an appropriate indicator of the PSII photochemical yield and can be used to monitor the physiological state of adherent cell cultures of the benthic dinoflagellate *P. belizeanum*.

### 2.3. Synthesis of Pigments

**Figure 7:**
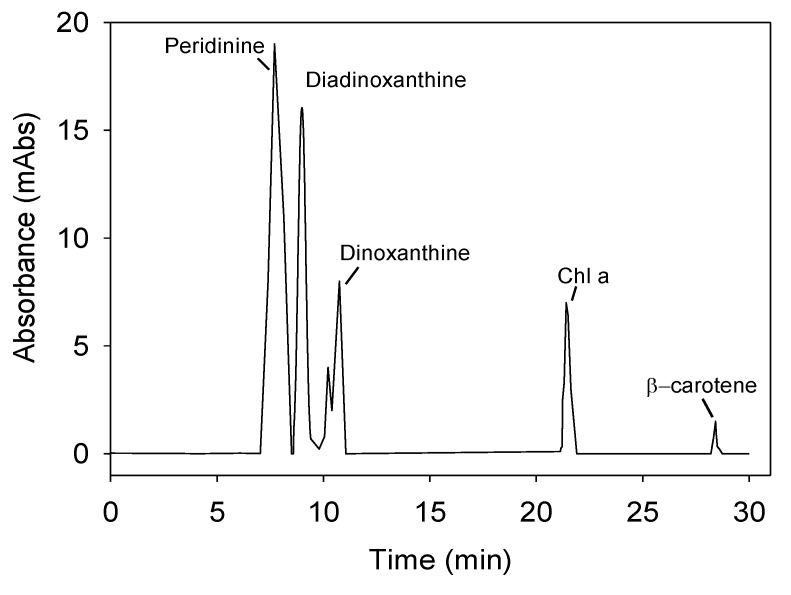
Representative chromatogram of the pigment profile of *P. belizeanum*, obtained from a methanol extract by means of a diode-array HPLC system. Chlorophyll *c* was determined spectrophotometrically as indicated in Experimental Section 3. The cells used came from an inoculum culture.

Figure 7 shows a representative chromatogram of the pigment profile of *P. belizeanum* measured by high performance liquid chromatography (HPLC) in the culture used as inoculum. The following pigments were identified in *P. belizeanum* cells: chlorophyll *a* (Chl *a*) chlorophyll *c* (Chl *c*), β-carotene, diadinoxanthin (*Dd*), peridinin (*Pe*) and dinoxanthin (*Dn*). As the β-carotene concentration in the cells was always very low and was not detected in most of the chromatograms, it was excluded from the next analysis. 

The abundance of carotenoid pigments (*Dn*, *Dd* and *Pe*) for different species of the genus *Prorocentrum*, expressed as molar ratios with respect to Chl *a*, are compared in Table 4. In general, intervals of variation of these ratios obtained in this study for *P. belizeanum* were consistent with the values reported for other species of the same genus, though some of the maximum and minimum values for *P. belizeanum* were slightly higher and lower, respectively. This was likely due to the different aforementioned stressful *T*-*I*_0_ combinations that could give rise to marked changes in pigment cellular content, as will be discussed below. 

**Table 4:** Intervals of variation of molar pigment ratios to Chl *a* in the genus *Prorocentrum* extracted from [37] for comparison with those observed in our study for *P. belizeanum*.

Ratios	Level	From [37]		Our study	
		Strain	Content	Strain	Content
Perid/chl *c*	Max	*P. levis*	38.92	*P. belizeanum*	43.80
	Min	*P. nux*	3.51		1.18
Perid/chl *a*	Max	*P. rostratum*	1.69		2.08
	Min	*P. arenarium*	0.95		0.06
Chl *c*/chl *a*	Max	*P .nux*	0.43		0.19
	Min	*P. levis*	0.03		0.14
Diadino/chl *a*	Max	*P. lima*	1.09		1.36
	Min	*P. minimum*	0.43		0.07
Dino/chl *a*	Max	*P. lima*	0.3		3.70
	Min	*P. minimum*	0.07		0.06

Table 3 presents results of a multifactor ANOVA carried out with all the pigment data measured during culture. Temperature was responsible for most of the variation observed in the pigment profile of *P. belizeanum*, while the influence of *I*_0_ was smaller. The interaction *T*-*I*_0_ only had a statistically significant effect (*p* < 0.05) on *Dn*, but its contribution to the overall variation of intracellular pigments was considerably inferior to the sum of *T* and *I*_0_ single factors. The remaining interactions were not significant. 

**Figure 8:**
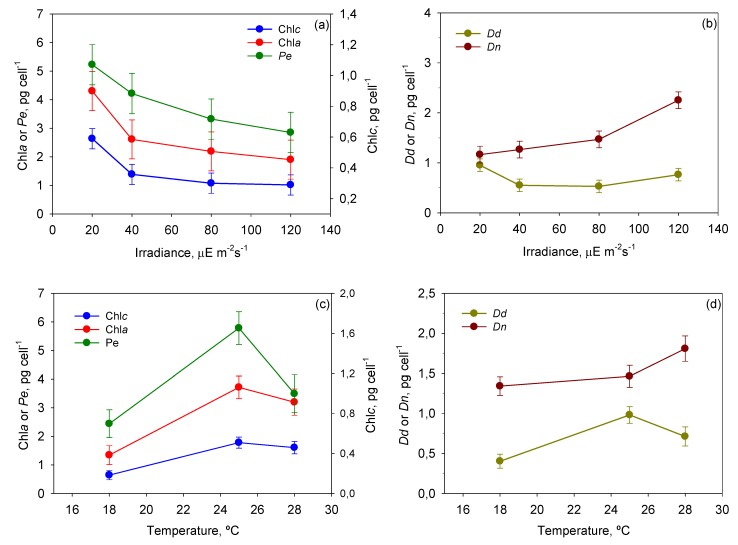
Evaluation from the ANOVA of the effect of *I*_0_-*T* on the pigment profile of *P. belizeanum*. Every irradiance and temperature gathers all the results obtained for the three *T* and four *I*_0_ levels, respectively. Bars around points represent 95.0% confidence intervals based on Fisher’s least significant difference (LSD) procedure. Overlapping bars indicate no significant difference. (**a**) Effect of *I*_0_ on Chl *a* and Peridinin; (**b**) Effect of *I*_0_ on Dinoxanthin and Diadinoxanthin; (**c**) Effect of *T* on Chl *a* and Peridinin; (**d**) Effect of *T* on Dinoxanthin and Diadinoxanthin.

Figure 8 shows the mean cellular content of each pigment, provided by the ANOVA, as a function of *T* and *I*_0_. Photolimited cells (<40 µE·m^−2^·s^−1^) strongly increased the content of light-harvesting pigments such as chlorophylls and peridinin (*p* < 0.05) in relation to photoinhibited cells (120 µE·m^−2^·s^−1^). The most visible change was the darkening of cells, which is observed in all photosynthetic microalgae grown under dim light [38]. The same pigments decreased under high irradiance, resulting in cells being rather transparent. The opposite was observed with the photoprotective pigments of the xanthophyll cycle (*i.e.*, diadinoxanthin and dinoxanthin). Although *I*_0_ had no statistically significant influence on *Dd*, the pool of *Dn* + *Dd* did increase when cells were exposed to high irradiance levels. This augmentation was concomitant with the drop in *F*_v_/*F*_m_ at irradiances above 40 µE·m^−2^·s^−1^ (see Figure 4). This pattern is part of a vital mechanism of coping with excess irradiance by phototrophs that have been extensively described in microalgae over recent decades and is consistent with our results. Xanthophyll-cycle pigments are accumulated by the cells to increase thermal energy dissipation as photon harvesting rates exceed those required in photochemistry. 

There are numerous studies, particularly with dinoflagellates, concerning the effect of temperature on the synthesis of pigments in microalgal cells [39,40,41,42,43,44]. In our study, *P. belizeanum* increased its content of chlorophylls and carotenoids with temperatures up to 25 °C (Figure 8c,d). *Dn* quota continued to grow up to 28 °C (*p* < 0.05), but peridinin and diadinoxanthin were exceptions under thermal stress at 28 °C. Their content decreased significantly (*p* < 0.05), though always maintaining a value above that registered at the lowest temperature assayed (18 °C). The tendency of chlorophylls is in accordance with results previously reported for several microalgal species [39,40,41,42,43], suggesting a common pattern of decrease in maximum rate of energy consumption with increasing temperature, as indicated by Carvalho *et al*., [45]. The tendency of the carotenoids may be partially explained by reactive oxygen species (ROS)-mediated stimulation of carotenogenesis as reported with *Haematococcus* spp [42]. However, the decrease in *Dd* at the highest temperature (28 °C) is a particular phenomenon, which is only observable, as far as we know, in a few species of symbiotic dinoflagellates [44]. The fundamentals lies on that an excessive ROS production due to elevated temperature, with or without high irradiance, may trigger the selective destruction of photosynthetic pigments by photo/thermal oxidative processes in symbiotic dinoflagellates [46,47]. Thus, the dinoflagellate *Symbiodinium* is able to interconvert the pigments *Dd* and diatoxanthin (*Dt*) as part of a photoprotection process, which is vital to the prevention of ROS overproduction [48]. More recently, it was observed in several symbiotic algae that elevated temperature/irradiance caused increased levels of *Dt* in relation to the *Dd* + *Dt* pool due to the preferential destruction of *Dd*, without a significant impact on chlorophyll *a* [44]. In our results, the role of dinoxanthin (*Dn*) in *P. belizeanum* at 28 °C (see Figure 8c,d) seems to match that of the diatoxanthin in other dinoflagellates [44]. Further research is, nevertheless, required in order to clarify this issue fully in non-symbiotic dinoflagellates.

### 2.4. Okadaic Acid Production

OA and analogues are secondary metabolites and therefore their synthesis is completely uncoupled from cell growth. They are mainly accumulated under long-term starvation. For this reason, determination of OA in cells was carried out in the stationary phase of growth. Cellular content of OA and its concentration in the broth were accordingly calculated and a multifactor ANOVA was performed (see Table 3). Temperature had a statistically significant effect on both OA cell content and concentration (*p* < 0.05), whereas the effect of irradiance on these responses was much less, proving irrelevant for OA concentration. 

**Figure 9:**
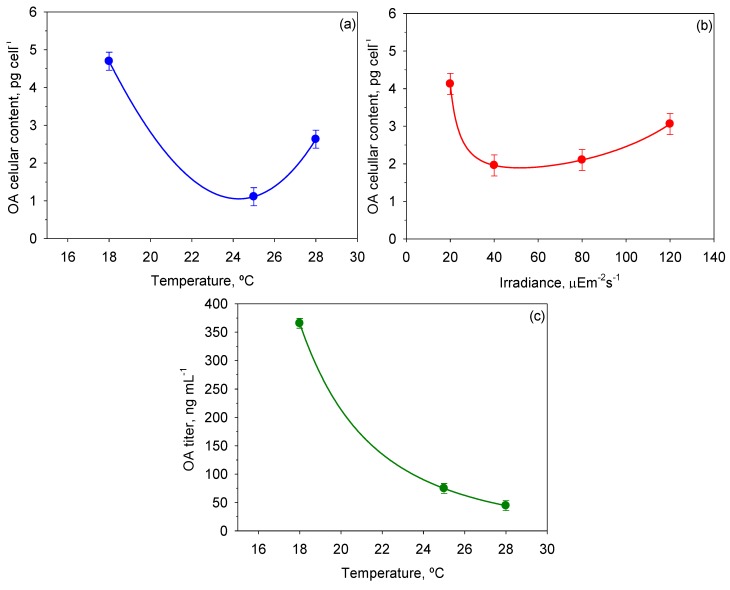
Effect of *I*_0_ and *T* on OA cellular content and okadaic acid (OA) titer in cultures of *P. belizeanum*. Every irradiance and temperature gathers all the results obtained for the three *T* and four *I*_0_ levels, respectively. Bars around points represent 95.0% confidence intervals based on Fisher’s least significant difference (LSD) procedure. Overlapping bars indicate no significant difference. (**a**) Effect of *T* on OA cellular content; (**b**) Effect of *I*_0_ on OA cellular content; (**c**) Effect of *T* on OA titer.

Figure 9 shows the mean OA cellular content and OA titer values reached for each *T* and *I*_0_ and the intervals around each mean value. Inspection of Figure 9a indicates minimal accumulation of OA in cells at the optimal growth temperature, and maximum accumulation at the lowest temperature. According to Figure 9b, only the lowest *I*_0_ had a statistically significant influence on OA content in cells. On the other hand, the OA titer in the broth dropped abruptly with the temperature (see Figure 9c). Thus, the maximum values of 6.85 ± 0.61 ng cell^−1^ and 419.0 ± 12.2 ng mL^−1^ for OA content per cell and OA titer, respectively, were obtained at 18 °C and 20 µE·m^−2^·s^−1^ (data not shown). OA production in *P. belizeanum* seems to be a response to stressful environmental conditions, as the OA content increased in two different circumstances: (i) with both low *T* and low *I*_0_, when growth is limited; and (ii) with high *T*, when growth is strongly inhibited. Presumably, synthesis of OA and other toxins in dinoflagellates may be an innate defence mechanism to suppress the growth of potential competitors [49], which is accentuated when growth is acutely stressed. 

The above-described pattern partially resembles the observations reported for other species of the genus *Prorocentrum* [50]. Apparently, *P. belizeanum* produced less OA than others of the same genus. OA cellular contents have been reported to range from 0.5 pg cell^−1^ to 192 pg cell^−1^, though large variability due to environmental factors is reported [50]. However, the importance of this strain does not lie solely in the production of OA; OA analogues (lipophilic dinophysistoxins (DTX1–3) and the water-soluble derivatives (DTX4 and DTX5a–c)) and linear polycyclic compounds and macrolides (e.g., corozalic acid, belizeanolide, belizeanic acid) are also of great interest [7,8,9,10,11,12]. Unfortunately, the monitoring of the synthesis of these metabolites in culture is not possible, since the corresponding reference standards are not commercially available, and/or a routine laboratory-scale methodology for determining them has not been reported. Meanwhile, the determination of OA can serve as a reference for the other analogues and polyketides. The rationale for this lies in recent evidence, comprehensively reported by Hu (2010), on the biosynthetic origin and bioactivity of OA analogues [13]. Although different *Prorocentrum* species can produce a great variety of different OA derivatives with different substituents or conformational change on their skeleton [13], these toxins seem to be biosynthesized via a polyketide pathway shared with OA. Therefore, the more than forty compounds identified to date from a reduced number of species of the genus *Prorocentrum* [13] may be indirectly monitored in culture through the determination of OA. This approach would have a clear benefit, since it would allow the planning of inexpensive experiments aimed to optimize culture conditions for enhancing the production of the toxins. Large-scale cultures could then be designed to obtain sufficient amounts of compound to carry out all the necessary studies. Nonetheless, a possible metabolic inter-conversion between toxins due to the effect of culture conditions should be taken into account in subsequent studies, because it can induce different ratios of OA and other analogues. This was the case of *Prorocentrum lima* with OA and methyl-okadaic acid [50].

### 2.5. Implications in Massive Culture

Dinoflagellates and their toxins are generally hazardous if inhaled or ingested. Safety issues merit special attention when producing dinoflagellates and toxins thereof, even more so as the scale of operation increases. A comprehensive safety management plan is necessary for any commercial facility producing or processing biotoxins [1]. If producer species are photosynthetic, the costs of implementation of that plan can be greatly reduced because they may be grown in closed photobioreactors under outdoor conditions. However, excessive irradiance can be detrimental for cells. 

At irradiances beyond the maximum regions represented in Figure 3a and Figure 4a, PSII can be strongly photoinhibited, so bioproductivity will decrease rapidly as *I*_0_ is further raised. Therefore the previous determination of the photoinhibition threshold of any photosynthetic microalgal species is crucial to the reliable design of an outdoor culture system. The tolerance to light flux of *P. belizeanum* was extremely low (around 50 µE·m^−2^·s^−1^), indicating that it is a light-sensitive species. Other microalgal species successfully grown in outdoor conditions could stand the local irradiance levels that occur in moderately sunny areas of the planet (e.g., up to 3000 µE·m^−2^·s^−1^ in Southeast Spain). Consequently, photobioreactors for growing them must be modified accordingly to prevent photoinhibition and to optimize the photosynthetic efficiency of the cells. Among the diverse strategies that may be devised to address this issue, the following may be mentioned aimed at attenuating the incident radiation on the surface of the photobioreactor: (i) covering the photobioreactor with meshes of different density depending on the degree of required illumination reduction; (ii) using dyed material for the construction of the photobioreactor (dyes should be carefully selected to avoid distortions in the spectrum of the photosynthetically active radiation (PAR)); or (iii) increasing the height of the column of water that circulates over the surface where the cells are attached. Not all dinoflagellates are equally sensitive to light; intra- and inter-specific differences exist [5,51]. Therefore, if a product can be sourced from multiple species, the selection of a more resistant species to outdoor culture conditions may have important advantages for large-scale production. Enhancements in the concentration of a target compound may also be made by improving the producer strain through genetic modification. Unfortunately, the molecular genetics of the biosynthesis of dinoflagellate toxins is poorly understood, though progress is being made and discussed [3].

**Figure 10:**
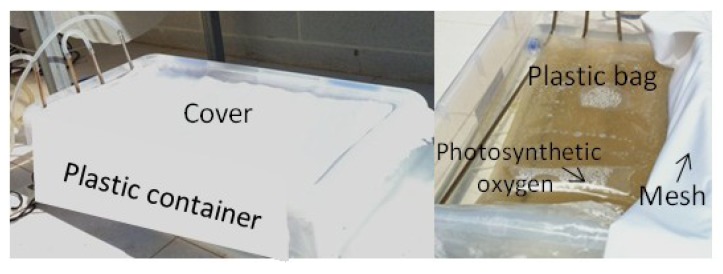
Photographs of an unsophisticated bench-scale outdoor culture for testing photoinhibition of *P. belizeanum* under solar illumination.

According to the results presented here, synthesis of OA (and probably derivates thereof) in *P. belizeanum* seems to be stimulated under stressful temperature and/or illumination conditions. In a hypothetical outdoor culture, cells would be exposed to daily changes in irradiance and temperature. By properly controlling these two factors over time, we may culture *P. belizeanum* in a two-step process to increase the OA yield (*i.e.*, cyclically alternating a period of optimal culture conditions with one of stressful conditions in which a part of the biomass is harvested to recover OA and the other is left in the photobiorreactor to begin a new cycle). Figure 10 shows photographs of a rudimentary bench-scale outdoor culture with *P. belizeanum* operated in batch mode. The culture system consisted of plastic bags covered by a mesh during the midday to reduce the irradiance at the surface of the culture to below 80 µE·m^−2^·s^−1^. Temperature was controlled around 18 °C. Outdoor culture reached 25,000 cells·mL^−1^ after 10 days. The results were consistent with the experiments performed in T-flask at the same temperature and similar irradiance (see Figure 1d). From that day, the culture was partially uncovered, reaching irradiance levels of up to 320 µE·m^−2^·s^−1^ on the bag surface. The cells were strongly photoinhibited and in the course of the next 10 days over 50% of the cells died. 

## 3. Experimental Section

### 3.1. Species Selected

The strain used in this study was a clone of the photosynthetic toxic benthic dinoflagellate *Prorocentrum belizeanum* (VGO1029), kindly donated by the Culture Collection of Harmful Microalgae of IEO (Vigo, Spain) and already adapted to grow in L1 medium. *P. belizeanum* is commonly associated with floating detritus and sediments in tropical bays of the Caribbean Sea [45]. *P. belizeanum* is a known producer of okadaic acid and derivatives thereof [8,11], dinophysistoxin-1 [14] and cytotoxic macrolides [10].

### 3.2. Culture and Growth Kinetics

**Figure 11:**
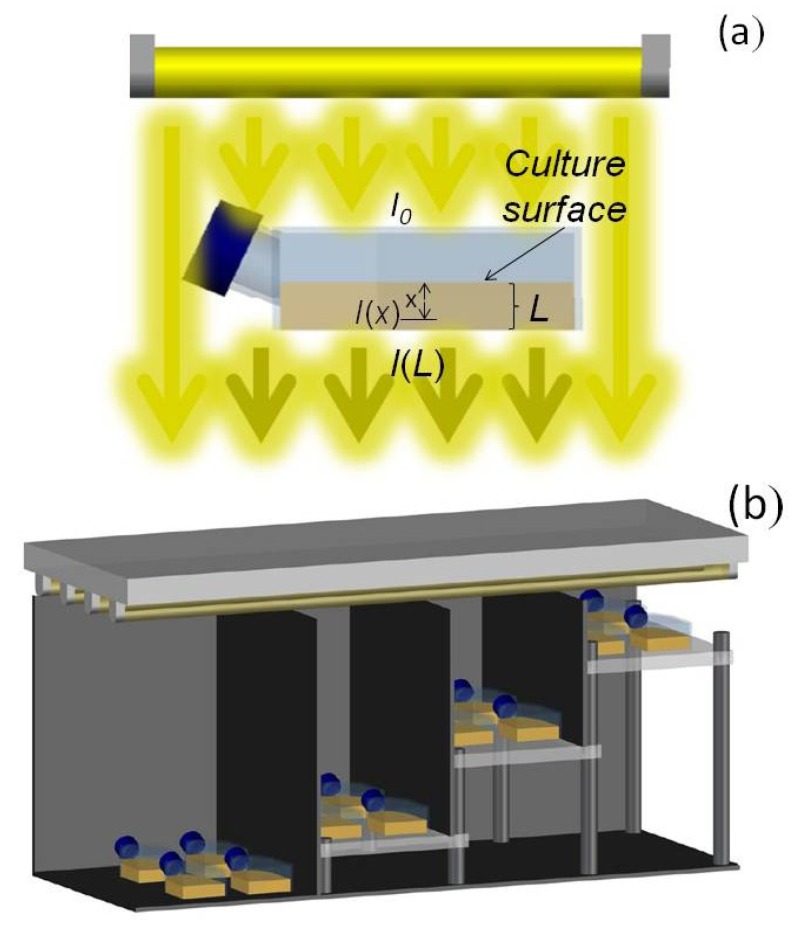
Diagram of the culture system. (**a**) Attenuation of irradiance *I*_0_ in a T-flask with culture thickness *L*; (**b**) Arrangement of T-Flasks within the illumination system.

Basal inocula were grown under a 12:12-h light-dark cycle (100 μEm^−2^s^−1^) at 18 ± 1 °C. Filter sterilized (0.22 mm Millipore filter; Millipore Corporation, Billerica, MA, USA) L1 medium [52] prepared in natural Mediterranean Sea water (conductivity around 55,000 µS/cm) was used in all experiments. Preliminary experiments showed that reproducible and consistent growth occurred when cultures were maintained in vented polystyrene tissue culture T-flasks with vent/close cap (Nunclon^®^ Δ surface treatment). As *P. belizeanum* is a benthic dinoflagellate microalga, cells grew forming numerous lumps consisting of dozens of cells that adhered to the T-flask bottom. In addition, cells secreted an extracellular mucus that covered the bottom forming a thin transparent layer. Cell confluence was not observed in any experiment. Culture T-flasks were horizontally located on an incubator tray and illuminated overhead by a bank of cool daylight fluorescent tubes (Philips TL40W). T-flasks of parallelepipedal geometry (see Figure 11a) were chosen because of the ease in the evaluation of the light field inside the cultures as reported elsewhere [53]. The fixed culture volume (15 mL) provided a high surface to volume ratio, and thus a relatively low culture thickness. It is a relevant issue to be tackled prior to planning experiments for obtaining growth curves as a function of irradiance. Many factors, such as light scattering effects and/or mutual shading (when cells close to the illuminated surface intercept more light that they can use, and thereby shade algae deeper in the culture), may lead to a misevaluation of the effect of irradiance due to overestimation of the light available to cells. One way to avoid or mitigate this phenomenon is to work with optically thin cultures with minimum mutual shading. Our cultures fulfilled this condition because they had a high surface to volume ratio that provided a short light path (*L*) through the cultures. The rationale of this is based on the following analysis. Since the attenuation of any given light beam traveling through the medium can be calculated by the Lambert-Beer’s law [53], irradiance at any inner point separated by distance *x* from the culture surface (see Figure 2a) is obtained as:

*I(x) = I_0_e^-xKN^* (1)

where *I_0_* represents the irradiance measured on the culture surface, *N* is the cell concentration and *K* the extinction coefficient of the cells. Therefore, if a culture of thickness *L* is optically thin, the term *LKN* approaches zero and then from Equation (1) it is derived that *I*(*L*) ≈ *I*_0_. To verify that this condition was satisfied during the whole culture time, the irradiance that left the bottom of the T-flask, *I*(*L*), was regularly measured, subtracting the attenuation due to the culture medium, and the relative irradiance attenuation, (*I*_0_ − *I*(*L*))/*I*_0_, was calculated as an indicator of the mutual shading.

Four irradiance levels (20, 40, 80, and 120 µE·m^−2^·s^−1^) at three temperatures (18, 25, and 28 °C) were assayed. Cultures were inoculated into 25 cm^2^ T-flasks containing 15 mL of L1 medium. The light:dark cycle and light source were the same as those described above for the inocula. To minimize undesirable effects on growth kinetics and toxin production due to possible photo- and thermo-acclimation phenomena in the inoculum, cultures were pre-acclimated to every experimental *I*_0_*-T* combination by repeatedly subculturing over a period of 4 weeks. All T-flasks were placed in an incubator as shown in Figure 11b. The walls were painted black to avoid reflections of the light that might modify the irradiance distribution on the surface of the cultures and thereby the illumination field in the cultures. The different irradiance levels were provided by varying the distance from the T-flask surface to the light source. Irradiance was measured with a 4π sensor (Biospherical instruments Inc., mod. QSL-100, San Diego, CA, USA). Experiments were carried out in duplicate. 

Growth was measured by cell counts. For sampling, the cells were gently detached with cell scrapers to prevent cell breakage. The culture was then mildly agitated by hand to distribute uniformly cells in the culture. Afterwards, 1-mL samples were collected daily, fixed with lugol’s solution (L6146, Sigma-Aldrich, Co., Saint Louis, MO, USA), and the cells were counted on a Sedgewick-Rafter counting slide. This sampling technique was the least invasive of all tested. In fact, the cultures returned to the same original appearance hours later (data not shown).

The batch cultures of *P. belizeanum* exhibited asymmetric growth curves, *i.e.*, a sharp decline in cell density was observed following the stationary phase. As this behavior cannot be described by the standard (symmetric) logistic equations, the following asymmetric logistic equation (ALE) was used to model cell concentration *versus* culture time data. In this way the corresponding specific growth rates were easily obtained by analytical differentiation of the ALE:

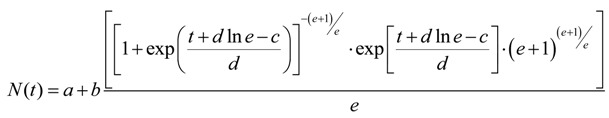
(2)
where *a*, *b*, *c*, *d* and *e* are fitting parameters. As reported elsewhere [54], this type of equation is used as a mathematical tool to reproduce raw experimental results, making possible the explicit calculation of their values and the derivatives thereof. Thus, the cell-specific growth rate *µ* (days^−1^) was calculated as a function of the culture time as follows:

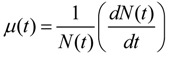
(3)


A previous discrimination analysis of equations using the F-test proved that the fits of Equations (2) and (3) were statistically superior to the ones with other equations at the 95% confidence level. 

### 3.3. Modelling of Growth

The following mechanistic model of photosynthesis in microalgae (*P*-*I* curve) proposed by Camacho *et al*. (2003) [27] for nutrient-replete cells has been used to model the effect of the irradiance:

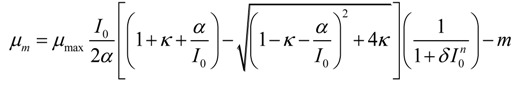
(4)
where *µ*_m_ is the maximum specific growth rate in a certain experiment and *µ_max_*, *α*, *κ* and *δ* are fitting parameters of the model that have rigorous biological meaning [27]. The non-dimensional group *α*/*I* represents the ratio between the maximum rate of photosynthesis and the energy absorbed. The parameter *κ* represents the fraction of the activated PSUs that give rise to a fractional productivity of 0.5 (PSU is a photosynthetic unit of the cell, *i.e.*, a portion of the thylakoidal membrane that brings together photon receptors, electron carriers, and the enzymes necessary for generating NADPH and ATP). The parameter *δ* represents the ratio of the kinetic constants for photoinhibition and repair processes in the cells. The constant *n* represents the reaction order in irradiance during the process of light-induced damage to photosystem II in the PSU. Parameter *m* is the cellular specific maintenance rate.

The effect of the temperature (*T*) on microalgal growth has been simulated with the following empirical equation, not based on the Arrhenius equation, recently proposed by Huang *et al*. (2011) [28] for descriptive microbiology:

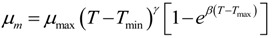
(5)
where *T_min_* and *T_max_* are parameters defined as the minimum and maximum temperatures, respectively, at which the growth rate is zero; *γ* and *β* are constants of the model with physiological meaning.

### 3.4. Measurements of the Chlorophyll Fluorescence

Long-term kinetics of chlorophyll fluorescence in *P. belizeanum* were recorded with a pulse amplitude modulation (PAM) chlorophyll fluorometer (Mini-PAM-2500). All samples were kept in darkness for 30 min before measurements in order to determine photosynthetic parameters in a dark-adapted state. The initial fluorescence (*F*_0_) was measured by exposing cells to a red light weak pulse (<1 μEm^−2^s^−1^). Maximum fluorescence (*F*_m_) was then quantified by applying a saturating light pulse (8000 μEm^−2^s^−1^; 800 ms pulse-width). The maximum variable fluorescence was calculated as *F*_v_ = *F*_m_ − *F*_0_. The *F*_v_/*F*_m_ ratio, designated as the maximum photochemical yield of the photosystem II (PSII), is unanimously considered an indicator of cell stress since it represents the performance of photochemical processes in the PSII. 

### 3.5. Outdoor Cultures

A bench-scale outdoor photobioreactor was custom built to a design based on previous experience with the T-flasks. The bioreactor was made with custom-made polyethylene bags (32 cm × 65 cm), which were introduced in parallelipepidal structure containing water. The culture temperature was controlled at 19 ± 1 °C by circulating thermostated water. The working volume was 18 L. The photosynthetically active irradiance on the surface of the photobioreactor and in the centre of the bag was measured using a QSL-100 quantum scalar irradiance sensor (Biospherical Instruments, San Diego, CA, USA). To reduce incident irradiance, the photobioreactor was covered with white fabric. Thus, irradiance on the bag surface ranged from 80 to 320 µE·m^−2^·s^−1^. Bags were partially sterilized using a microwave oven (2 min, 800 W). The reactor was then filled with the L1 medium made in filter sterilized Mediterranean Sea water. Inoculum was in the late exponential growth phase. The cell concentration in the freshly inoculated bioreactor was about 10,000 cells mL^−1^, and the photobioreactor was operated in batch mode.

### 3.6. Identification and Quantification of Pigments

Cell-free methanol extracts were obtained from culture samples as described elsewhere [55]. Detection of pigments in methanol:H_2_0 (9:1) extracts was performed by HPLC with a diode array detector as described in [56] using a Shimadzu SPD-M10AV high-performance liquid chromatograph (Shimadzu Corporation, Kyoto, Japan). Separation was performed on a Lichrosphere RP-18 5 μm column (4.6 × 150 mm). The eluents used were (A) water/methanol (2:8, *v*/*v*) and (B) acetone/methanol (1:1, *v*/*v*). The pigments were eluted at a rate of 1 mL/min and detected by measuring absorbance at 360–700 nm using a diode array detector. Standards of β-carotene and chloropyll *a* were provided by Sigma Chemical Co. (St. Louis, MO, USA), and peridinin, diadinoxanthin and dinoxanthin were from DHI Water & Environment (Horsholm, Denmark). Chlorophyll *c* content was determined by Hansmann’s spectrometric method [57] and using Parsons and Strickland’s equations for the quantification [58].

### 3.7. Determination of OA Concentration

Okadaic acid was measured separately in the biomass and in the culture supernatant following the method of Kelly *et al*., [59]. OA was recovered from samples by chromatography on silica (Sep-pak Plus C18. Waters, Milford, MA, USA) for the biomass extracts (MeOH) and Diaion^®^ HP-20 (glass columns packed with 500 mg of HP-20 from Supelco) for supernatants. Using 1-bromoacetylpyrene (BAP), as derivatization reagent, the OA was determined by HPLC with fluorimetric detection. An HPLC system (Shimadzu AV10; Shimadzu Corporation, Kyoto, Japan) with a fluorescence detector (Shimadzu RF-10AX), an auto-injector (Shimadzu SIL-10ADVP) and a Supelcosil^TM^ LC-18 column (25 cm × 4.6 mm, 5 µm. Supelco) were used. The reference standard used was okadaic acid sodium salt (sodium okadate) provided by CIFGA (Cifga S.A., Lugo, Spain).

### 3.8. Statistical Analyses

Nonlinear regression to fit specific growth rate data to Equations (4) and (5), and multifactor ANOVA tests were conducted in STATGRAPHICS Centurion software package (StatPoint, Herndon, VA, USA). 

## 4. Conclusions

This study has demonstrated that the benthic dinoflagellate *P. belizeanum* is a moderately mesophilic and extremely light-sensitive species. Cells showed a pronounced up-regulation of the pool of protective pigments of the xanthophyll cycle (diadinoxanthin and dinoxanthin) to dissipate excessive photonic energy as heat. Furthermore, *P. belizeanum* also reduced the content of peridinin, a light-harvesting pigment, at high irradiance/temperature; likely cells tried to minimize excessive light-absorption under stressful environmental conditions. However, these mechanisms of photoprotection were insufficient to prevent apparent photoinhibition above 40 µE·m^−2^·s^−1^ and thermal stress at 28 °C. A continuous sharp drop in *F*_v_/*F*_m_ during culture was the prelude to this metabolic response, which finally led to death of the culture under the most stressful *T*-*I*_0_ combination (120 µE·m^−2^·s^−1^ and 28 °C). Chlorophyll *a* fluorescence thus represents a powerful technique, which allows rapid monitoring of physiological status in dinoflagellate culture, but always accompanied by other cellular responses (e.g., pigment profile).

T-flask culture observations were consistent with preliminary assays carried out in outdoor conditions, reinforcing the above-mentioned considerations to be taken into account before a hypothetical mass cultivation of this species. OA overproduction was a consequence of stressful environmental conditions owing to the combination of low temperature and irradiance, or due to high temperature alone.
